# Normonatremic Transient Renal Salt Wasting (TRSW) Is Not Rare in a Department of Internal Medicine

**DOI:** 10.3390/jcm12020397

**Published:** 2023-01-04

**Authors:** Wim Musch, Guy Decaux

**Affiliations:** 1 Department of Internal Medicine, Iris South Hospital, B-1070 Brussels, Belgium; 2 Research Unit for the Study of Hydromineral Metabolism, Department of Internal Medicine, Erasme Hospital, Free University of Brussels (ULB), B-1070 Brussels, Belgium; 3 Department of Internal Medicine, Molière-Longchamps Hospital, Rue Marconi, 142, B-1190 Brussels, Belgium

**Keywords:** salt wasting, calciuria, stroke, angina pectoris, natriuric factors, kaliuria

## Abstract

Background: We previously reported that for around 5% of patients hospitalized with hyponatremia, it was related to what is called “transient renal salt wasting” (TRSW). In the present study we ask whether TRSW can also be observed in patients without hyponatremia. Methods: In this observational retrospective study we analyze the urine solute excretion of 200 consecutive normonatremic patients with normal kidney function and admitted in our department over one year. Patients were selected for analyses of FE.K, UCa/UCr and FE.PO_4_ if FE.Na was higher than 2% (N < 1.6%) before any treatment, and only if they were not taking diuretics. Result: Eleven normonatremic patients presented with transient high FE.Na > 2% on admission (2.9 ± 0.6% with a high FE.K of 28 ± 6.4%; a high UCa/UCr of 0.37 ± 0.13 and a high FE.PO4 of 23.2 ± 9.6%). All of these patients were elderly. Seven were female and four were male. Neurological disorders were observed in six patients (three strokes, one transient ischemic attack, one syncope and one epileptic attack). Heart problems were observed in three patients (all angina pectoris, two of which also had HBP). One patient presented with rectal bleeding with HBP, and another presented COPD with a pneumothorax. One patient with angina pectoris showed a transient relapse after four days of hospitalization (FE.Na 3.6%). The urine electrolyte excretion in these patients are similar to those observed after furosemide intake. Conclusion: Normonatremic TRSW is not a rare observation, particularly in patients with neurological or cardiac problems.

## 1. Introduction

Usually we pay little attention to urine electrolyte excretion in patients without abnormality in serum electrolytes. However, when there is a serum electrolyte disorder, we know that urine electrolyte measurement is essential for the diagnosis. We previously reported a series of 110 consecutive hyponatremic patients of different origins, and observed six patients [1] in whom hyponatremia was due to a transient high renal solute loss (TRSW). In these patients, the administration of isotonic saline corrected the hyponatremia [1,2]. These patients were not taking diuretics, did not present an osmotic diuresis, did not receive toxic agents such as cisplatin, and did not have a mineralocorticoid deficiency or an intrinsic nephropathy [2]. These patients also did not present any brain disease (that could induce what is called cerebral salt wasting (CSW)) [3,4,5].

Debate continues about the existence and true prevalence of the syndrome of cerebral/renal salt wasting. The suggested pathophysiology in these patients is the release of yet unidentified circulating factors that inhibit the reabsorption of Na^+^ and of urate in the proximal collecting tubule. In few reported cases, the FE.Urate remained elevated in patients who were thought to have cerebral/renal salt wasting after correction of hyponatremia with infusion of isotonic saline that resulted in the excretion of diluted urine [6]. Hence it was suggested that the FE.Urate after correction of hyponatremia may differentiate patients with SIADH from those with CSW/RSW [6]. It has been shown that, in patients with hyponatremia due to RSW, we also frequently have a high natriuria (FE.Na > 1.6%), a high kaliuria (FE.K > 20%), a high calciuria (FE.Ca > 2% or UCa/UCr > 0.20) and high phosphaturia (FE.PO_4_ > 20%) [1,2]. Here we describe that RSW can also be observed in patients without hyponatremia, and we show that not only are FE.Na very high (FE.Na > 2%) but also FE.K, FE PO_4_ and UCa/UCr. This transient high solute excretion is mainly seen in cardiac or neurologic diseases.

## 2. Materials and Methods

This is an observational retrospective study; over one year (2020) we analyzed the urine data of patients without hyponatremia and hospitalized in our Department of Internal Medicine before any treatment. This urine examination was systematically done at admission to any patient hospitalized in our unit before knowing the result of serum electrolytes. This was a habit to avoid wasting hours before requesting urinalysis when serum electrolyte results arrive in the unit. In this work we studied the records of patients with an abnormally high sodium excretion (FE.Na > 2%) (normal value of FE.Na is < 1.6%).

To be included, the patients did not have edema and ascites, nor overt cardiac, hepatic, or renal disease. The following serum and urine data were required: sodium, chloride, potassium, bicarbonate, calcium, phosphate, urea, creatinine, uric acid, glucose, and osmolality, as well as serum protein concentration and hematocrit (Hct). The spot urine sample allowed for the calculation of the fractional excretion (FE %) of filtered sodium, potassium, chloride, urea, uric acid and phosphate (FE.x = Ux/Px.PCreat/UCreat.100). We estimated the calcium excretion by the ratio UCa/UCreat. All the patients had to have a normal serum creatinine (<1.1 mg/dL).

We also compared the same parameters with patients who were taking furosemide.

This retrospective study was approved by the Ethics Committee of our hospitals (Ref. P2020/062).

## 3. Result

In our control group of patients of similar age and sex following a normal salt and solute intake, outside the hospital, the normal values for of FE.Na are 0.8 ± 0.4% and for FE.Osm 2 ± 0.5%.

**Figure 1:**
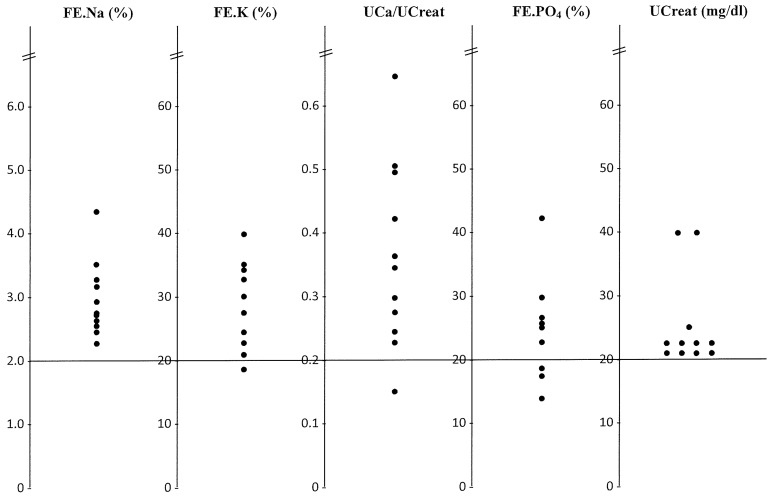
Patients with TRSL (transient renal sodium loss); UCa/UCreat represent the calciuria expressed per mg of creatinine, and diuresis is expressed by the urine creatinine concentration.

We arbitrarily selected to study all of the patients with a normal renal function (SCreat < 1.1 mg/dL) and a FE.Na > 2% (without diuretic intake). Eleven patients from the 200 patients where urine analyses were available are presented in Figure 1 and Table 1: three patients presented episodes of angina pectoris, three patients presented a stroke, one patient had atrial fibrillation and one a transient ischemic attack, HBP was present in three patients, syncope in one, epileptic crisis in one, rectal bleeding associated with HBP in one, and one patient with chronic pulmonary disease presented a pneumothorax. All of our patients were elderly (>65 y.o.) (see Table 1). Most of these 11 patients had other measurements of serum electrolytes in the days following their hospitalization. None had developed hyponatremia.

We had the opportunity with one patient to take many urine samples over four days (patient 8 of Table 1). At admission this patient presented angina pectoris associated with a FE.Na of 3.2% and a FE.K of 27.3%; eight hours later the FE.Na decreased to 0.59% and FE.K decrease to 8.8% (Table 2). After 48 h we observed a decrease in hematocrit from the initial value of 39.4% to 35.3%, while protein decreased from 7.1 g/dL to 6.2 g/dL. In this patient we noted again an increase in FE.Na (3.6%) the day before leaving the hospital. There was no cardiac complaining, and unfortunately no cardiogram was available. In this patient of Table 2 we also noted low value for Renin and aldosterone and a high value for ANP (185 ng/L; N 25–65 ng/L).

Two patients (see Table 1) presented an abnormally high serum protein concentration (patient n° 2 had a value of 8.4 g/dL and patient n° 4 had a value of 8.6 g/dL) (normal value ≤ 8.1 g/dL), one patient had an upper limit value of 8.1 g/dL.

Table 3 shows that our patients with TRSW presented similarities in solute excretion compared to those observed in patients treated chronically with furosemide during the first 4 h after 40 mg furosemide intake, while for five patients who were also treated chronically with 40 mg furosemide but for whom the urine samples were available just before the intake, we observed very low values, as expected.

## 4. Discussion

We were surprised to ascertain that, in a standardized approach of 200 consecutive hospitalized patients without hyponatremia, we observed eleven patients with a salt wasting state that was transient and not related to diuretic intake. The high different urine solute excretion observed in these patients is similar to those reported in previous hyponatremic patients with TRSW [1,2]. Our patients had normal GFR values for their age with a normal SNa, meaning that for a filtered load of around 12,000 mmol/day (140 × 0.060 × 1440), a 3% fractional excretion of Na would mean that 362 mmol Na would be lost should the disorder last for 24 h. Over a 4–8 h period, the loss would be 60–125 mmol, which is quite significant.

It is likely that if fluid intake had been larger some of the patients would have presented hyponatremia, or, if the duration of the high salt excretion had been longer and combined with higher fluid intake, hyponatremia would also have been observed. The mean U (Na + K) value of 134 mEq/l (slightly lower than the plasma sodium + potassium concentration) and the transient character of this disorder make the generation of hyponatremia unlikely, unless there is significant hypotonic fluid intake together with vasopressin activity. We believe that the data of the 11 described patients are consistent with the presence of one or several natriuretic agents [7,8,9,10]. Five patients presented upper limit of serum protein concentration (from 7.9 to 8.6 g/dL), supporting the hypothesis of initial volume concentration (see Table 1). We know that ANP/BNP-dependent decrease in blood pressure results in part from reduction in cardiac preload by causing the shifting of intravascular fluid into the extravascular compartment [7,8,9,10,11]. It was demonstrated that the intravenous injection of atrial extracts or ANP in rats induced high natriuresis, kaliuresis and urinary flow [11]. ANP also enhances renal calcium and phosphate excretion in rats [11] and hyperuricosuria can coexist with renal salt wasting in patients with intracranial disease [10]. Some of our patients presented high FE.Uric acid (>12%) (see Table 1). These data are consistent with our findings in our normonatremic TRSW patients, and it is likely that this could be explained by one or several undefined natriuretic agents, probably of the natriuretic peptide family.

A defect at the level of the Henle loop could also explain why some salt-losing patients have rather low initial urine osmolality, as observed in three patients (n° 1, n° 2 and n° 7). The high kaliuresis does not necessarily imply a distal tubular dysfunction, since the increase in sodium supply to the distal nephron could stimulate tubular secretion of potassium, accounting for the increase in potassium excretion. All of the normonatremic TRSW were observed in elderly patients. Most patients presented cardiac or neurological problems or high blood pressure. The high similarity in high transient sodium, potassium, calcium and phosphate excretion observed in our patients with the well-known early effect of furosemide on renal solute excretion must be noted.

Our data show that non-hyponatremic TRSW is not a rare observation. The mechanism of this TRSW stays to be established.

Unfortunately, this study is retrospective; a prospective study should be done on the usefulness of knowing the salt excretion obtained from a spot urine sample (to calculate the FE.Na or the UNa/UCreat ratio) in patients admitted to a hospital.

In a patient with atypical symptoms, the presence of a high FE.Na value (>2%) generally reflects the presence of a serious neurological pathology such as TIA, or a cardiac pathology such as angina pectoris. Further prospective studies are needed.

## 5. Conclusions

This section is not mandatory but can be added to the manuscript if the discussion is unusually long or complex.

## Figures and Tables

**Table 1:** Patients with TRSW (transient renal salt wasting).

Sex/Age	SNa	SK	Urea (mg/dL)	Creat (mg/dL)	Uric (mg/dL)	Ht (%)	Prot (g/dL)	Ca (mg/dL)	PO_4_ (mg/dL)	UOsm	FE.Na (%)	FE.K (%)	UCa/UCr	UCreat (mg/dL)	FePO_4_ (%)	FE.Uric (%)	
♂ 69	139	4.3	39	0.9	7.3	42	7.0	9.4	3.9	249	2.5	32	0.165	20	25.5	--	Angina
♀ 87	142	3.7	41	1	5.9	48.6	8.4	10	3.3	290	2.2	23	0.3	20	30	11	Angina/HBP
♂ 87	141	3.6	56	0.8	3.9	33.4	7.0	10.2	3.2	538	4.4	21.1	0.5	21	43	22.5	Rectal bleeding HBP
♀ 67	140	4.8	72	0.8	6.2	47.3	8.6	10.3	3.3	455	2.8	17.5	0.35	20	26.6	13	Syncope/HBP
♀ 84	136	4.2	23	0.6	--	40.1	--	9.2	3.6	422	2.7	25	0.64	21	27.5	--	Stroke
♀ 75	133	4.8	51	0.9	5.4	33.1	6.6	8.7	--	406	3.1	23	0.23	19	--	9.2	Epileptic crisis
♀ 77	140	4.1	35	0.9	4.6	37.6	7.9	10.1	2.9	320	2.4	35	0.30	20	18.6	14.7	Stroke
♀ 80	138	3.6	34	0.9	2.5	39.4	7.1	9.6	3.5	476	3.2	27	0.36	20	14	23.4	Angina
♀ 86	137	4.1	37	0.9	4.3	40.2	8.1	9.8	4.5	403	2.4	30	0.5	40	24	14.6	Transient ischemic attack
♂ 76	138	3.7	38	0.9	7.6	38.7	7.9	9.8	2.8	469	3.5	35	0.28	40	18	11	Stroke atrial fibrillation
♂ 68	140	4	34	0.7	4	--	6.1	8.7	2.8	394	2.6	39	0.43	26	--	15.5	Pneumothorax COPD

Normal values: FE.Na < 1.6%, FE.K ≤ 18%, UCa/UCr < 0.20, FE.PO_4_ < 20%.

**Table 2:** Eighty-year-old women hospitalized for an episode of angina pectoris—variations in different solute excretions are presented.

Angina	J0	J8hr	J24hr	J48hr	J96hr
	+	-	-	-	No clinical pain
PNa (mEq/L)	138		137	139	139
PK (mEq/L)	3.6		4.6	3.9	3.7
PUrea (mg/dL)	34		30	35	34
PCreat (mg/dL)	0.9		0.8	0.8	0.8
PUric acid (mg/dL)	2.5		3.2	2.7	3.2
Haematocrit (%)	39.4		39	35.3	35
PProtein (g/dL)	7.1		6.9	6.2	6.5
PCalcium (mg/dL)	9.6		9.6	8.5	9.1
PPhosphore (mg/dL)	3.5		3.5	3.8	4.2
FE.Na (%)	3.2	0.59	0.57	0.31	3.6
FE.K (%)	27.3	8.8	9.9	12	30
FE.Urea (%)	66	57	41	46	67
FE.Uric acid (%)	23.4	18	9.4	14	21
UCa/UCreat	0.36	0.25	0.13	0.14	0.2
Fe.PO_4_ (%)	14.4	14.8	11.4	10.7	21
UCr/PCr	22	44	50	50	25
UOsm	476	--	--	--	527
ADH (0–7 pg/mL)	0.6				
REN (7.5–40 pg/mL)	2				
Aldo (0–310 pg/mL)	52				
ANP (25–65 ng/L)	185				

Normal values: FE.Na < 1.6%, FE.K ≤ 18%, UCa/UCr < 0.20, FE.PO_4_ < 20%, FE.Urea < 55%, FE.Uric < 14%.

**Table 3:** Some serum and urine parameters in patients with transient renal salt wasting (TRSW) (*n* = 11) compared to patients with cardiac failure before (*n* = 5) and after early oral intake of 40 mg furosemide (*n* = 9).

Controls	TRSW (*n* = 11)	Under Furosemide (*n* = 9)	Before Furosemide (*n* = 5)
BW (kg)	63.6 ± 13	59.4 ± 11	NA
SNa (135–145 mEq/L)	138 ± 2.4	139 ± 3.6	138 ± 3
SK (3.5–5.1 mEq/L)	4.1 ± 0.45	4.1 ± 0.4	4.2 ± 0.3
Urea (17–48 mg/dL)	42 ± 12.5	46 ± 12	36 ± 17
Creatinin (0.7–1.1 mg/dL)	0.85 ± 0.1	0.9 ± 0.1	0.9 ± 0.1
FE.Osm (<3%)	5.1 ± 1.4	---	---
FE.Na (<1.6%)	2.9 ± 0.6	3.0 ± 0.8	0.23 ± 0.2 *
FE.K (<18%)	28 ± 6.4	22 ± 5.5	12.5 ± 5 **
UCa/UCr (<0.20)	0.37 ± 0.13	0.35 ± 0.12	0.10 ± 0.06 *
FE.PO_4_ (N < 20%)	23.2 ± 9.6	18.1 ± 6.2	11.5 ± 4.9

* *p* < 0.01; ** *p* < 0.05 (compared to early effect of Furosemide).
